# Inosine pranobex is safe and effective for the treatment of subjects with confirmed acute respiratory viral infections: analysis and subgroup analysis from a Phase 4, randomised, placebo-controlled, double-blind study

**DOI:** 10.1186/s12879-016-1965-5

**Published:** 2016-11-07

**Authors:** Jiří Beran, Eva Šalapová, Marian Špajdel, on behalf of the Isoprinosine Study (EWO ISO-2014/1) Team

**Affiliations:** 1 Vaccination and Travel Medicine Centre, Tylovo nábřeží 418/6, 500 02 Hradec Králové, Czech Republic; 2 Department of Epidemiology, 2nd Faculty of Medicine, Charles University, V Úvalu 84, 150 06 Prague, Czech Republic; 3 Ewopharma International, s.r.o., Hlavná 13, 831 01 Bratislava, Slovakia; 4 University of Trnava, Hornopotočná 23, 918 43 Trnava, Slovakia; 5 Institute of Normal and Pathological Physiology, Slovak Academy of Sciences, Sienkiewiczova 1, 813 71 Bratislava, Slovakia

**Keywords:** Immunomodulator, Immunosenescence, Influenza, Inosine pranobex, Isoprinosine, Viral infection, Efficacy, Safety

## Abstract

**Background:**

Inosine pranobex (Isoprinosine®) is an immunomodulatory drug approved in several countries for the treatment of viral infections. This study compared the efficacy and safety of inosine pranobex versus placebo in subjects with clinically diagnosed influenza-like illness, including subjects with laboratory-confirmed acute respiratory viral infections. Subgroup analyses evaluated the efficacy of inosine pranobex compared to placebo in otherwise healthy (without related ongoing disease) subjects that were less than 50 years of age and healthy subjects that were at least 50 years of age. The effect of body mass index (BMI) was evaluated in subjects less than 50 years of age.

**Methods:**

A total of 463 subjects were randomly assigned to receive inosine pranobex (*n* = 231) or placebo (*n* = 232) in this Phase 4, randomised, double-blind, multicentre study. The primary efficacy endpoint was time to resolution of all influenza-like symptoms present at baseline to none. Safety was evaluated through analysis of adverse events, vital signs, and physical examinations.

**Results:**

The difference in time to resolution of all influenza-like symptoms between treatment groups was not statistically significant but showed a faster improvement in subjects in the inosine pranobex group versus those in the placebo group - Hazard Ratio = 1.175; (95 % CI: 0.806–1.714). *P*-value = 0.324. In the subgroup analysis for subjects less than 50 years of age, statistically significant differences in time to resolution of influenza-like symptoms that favoured the inosine pranobex group over the placebo group were observed in those without related ongoing disease and those who were non-obese (BMI <30 kg/m^2^). The differences between the inosine pranobex and placebo groups in subjects at least 50 years of age without related ongoing disease and in subjects less than 50 years of age who were obese (BMI ≥30 kg/m^2^) were not statistically significant. Inosine pranobex was generally well tolerated, and no deaths were reported.

**Conclusions:**

The study results indicate the safety of inosine pranobex for the treatment of subjects with confirmed acute respiratory viral infections and confirm the efficacy of inosine pranobex versus placebo in healthy non-obese subjects less than 50 years of age with clinically diagnosed influenza-like illnesses.

**Trial registration:**

EWO-ISO-2014/1, EudraCT 2014-001863-11; Date of registration: 29 APR 2014; Detail information web link: https://www.clinicaltrialsregister.eu/ctr-search/trial/2014-001863-11/results

**Electronic supplementary material:**

The online version of this article (doi:10.1186/s12879-016-1965-5) contains supplementary material, which is available to authorized users.

## Background

Acute respiratory infection is a serious infection that is responsible for approximately 3.9 million deaths per year and is one of the leading causes of morbidity and mortality worldwide [1]. Acute respiratory infections are categorized as either upper or lower respiratory infections and are caused by well-recognised viral pathogens, including but not limited to influenza virus (types A and B), parainfluenza virus, respiratory syncytial virus (RSV), metapneumovirus (types A and B), coronavirus, rhinovirus, enterovirus, reovirus, bocavirus, and adenovirus, and bacterial pathogens, primarily *Streptococcus pneumoniae* and *Haemophilus influenza* [2, 3].

Influenza is an acute viral respiratory infection that affects 5 % to 15 % of the global adult population per year and results in approximately 0.25 to 0.5 million deaths and 3 to 5 million cases of severe illness worldwide annually [4, 5]. Influenza-like illnesses (ILI) are considered a subset of acute respiratory infections and result in the sudden onset of symptoms such as fever (body temperature greater than 38 °C), cough, and sore throat in patients [5]. Physicians have difficulty with the treatment of ILI because determining the aetiology is generally not possible solely on a clinical basis.

Current pharmacological interventions for the prevention and treatment of respiratory viral infections are primarily limited to vaccines and antivirals for influenza. Available antiviral treatments for influenza infection are M2 ion channel inhibitors (eg, amantadine and rimantadine) and neuraminidase inhibitors (eg, oseltamivir and zanamivir) [6]. If the infection is caused by bacterial pathogens, treatment can involve antibiotics and also medications that provide symptomatic relief. However, in case of viral aetiology, only medication for symptomatic treatment can be provided. [7].

Vaccination for seasonal influenza remains one of the standard approaches for prevention of the disease. However, immunisation rates for seasonal influenza remain low in many European countries even though the influenza vaccine is part of the national immunisation schedule in these countries [8]. Moreover, the vaccination is effective only when most of the circulating influenza viruses in a given season are similar to the viruses that were included in the influenza vaccine. The vaccine does not offer any clinical benefit against other pathogens that circulate during a season [9].

Inappropriate use of antibiotics for the treatment of acute respiratory infections has been observed during winter months, even though the majority of infections are caused by viral pathogens and are self-limiting. This practice may result in side effects and the development of antibiotic resistance in pathogens as well as an increased cost burden for the healthcare system [3, 10, 11].

Inosine pranobex (Isoprinosine®), a combination of the *p*-acetamidobenzoate salt of *N*,*N*-dimethylamino-2-propanol and inosine in a 3:1 molar ratio, is an immunomodulatory antiviral drug that has been licensed since 1971 in several countries worldwide for the treatment of viral infections [2, 12]. Inosine pranobex stimulates a nonspecific immune response that is independent of the specific viral antigen responsible for the ILI. In clinical studies, inosine pranobex has been shown to induce a type 1 T helper cell-type response in mitogen- or antigen-activated cells, and this response initiates T-lymphocyte maturation and differentiation and potentiates induced lymphoproliferative responses (13–15). Similarly, the drug modulates T-lymphocyte and natural killer cell cytotoxicity and CD8+ suppressor and CD4 + -helper cell functions and increases the number of immunoglobulin G and complement surface markers (14, 15). Inosine pranobex also increases cytokine interleukin (IL)-1 production and IL-2 production and upregulates the expression of the IL-2 receptor in vitro [13, 14]. The safety profile of inosine pranobex has been established through clinical trials for several indications and populations [2, 14–16]. A rapid increase in the number of mononuclear cells after the first dose of inosine pranobex was observed in 75 % of the subjects, and this increase was consistent with clinical observations of rapid resolution of common cold symptoms [15, 17].

This Phase 4 study aimed to compare the efficacy and safety of inosine pranobex with placebo in subjects with laboratory-confirmed acute respiratory viral infections in order to evaluate the clinical use of inosine pranobex for the treatment of acute respiratory viral infections. The primary efficacy endpoint was comparison between inosine pranobex and placebo groups in terms of the time to resolution of all influenza-like symptoms present at baseline to none. In a subgroup analysis of subjects with clinically diagnosed ILI, the study further evaluated the efficacy of inosine pranobex compared to placebo in healthy (without related ongoing disease) subjects less than 50 years of age and in those at least 50 years of age. The study also evaluated the effect of body mass index (BMI) in subjects who were less than 50 years of age and were non-obese (BMI <30 kg/m^2^) or obese (BMI ≥30 kg/m^2^). In addition, the study evaluated the efficacy of inosine pranobex in subjects less than 50 years of age for the time to resolution of all influenza-like symptoms present at baseline to mild or none (i.e. score of 1 or 0 on the influenza-like symptoms assessment scale).

## Methods

### Study design

This was a Phase 4, randomised, placebo-controlled, double-blind, multicentre study in subjects with clinically diagnosed ILI, including subjects with laboratory-confirmed acute respiratory viral infections due to influenza A or B virus, RSV, adenovirus, or parainfluenza virus 1 or 3. The study was conducted at 25 study sites in the Czech Republic (14 study sites) and Slovakia (11 study sites), with enrolment occurring between December 2014 and April 2015, and the last subject visit was on 03 June 2015. Detailed primary efficacy endpoints are provided in the Additional file 1.

### Inclusion criteria

Male and nonpregnant female subjects aged 18 to 75 years with a clinical diagnosis of ILI were included in this study. Influenza-like illnesses were defined as an oral temperature of at least 38 °C observed at the study site with at least 1 respiratory symptom of cough, sore throat, or nasal obstruction and at least 1 constitutional symptom of fatigue, headache, myalgia, or feverishness. The respiratory and constitutional symptoms were required to be considered by the subject as moderate or severe in intensity (a score of more than 1 on the 4-point influenza-like symptoms assessment scale). The subjects were required to have experienced the onset of ILI no more than 36 h prior to screening, where onset is defined as the time when the subject experienced fever and at least 1 respiratory symptom and at least 1 constitutional symptom. The full inclusion criteria and Influenza-Like Symptoms Assessment Scale are detailed in the Additional file 1.

### Exclusion criteria

Subjects were excluded from participation in this study if they met any of the following criteria: had an immunosuppressive disorder or were receiving immunosuppressive therapy; were undergoing treatment with xanthine oxidase inhibitors or uricosuric agents or treatment with thiazide or loop diuretics; had chronic renal dysfunction or severe liver function impairment; were lactose intolerant; had cancer in a nonremission stage; were undergoing treatment with zidovudine; were pregnant or lactating/breastfeeding; had received a dose of inosine pranobex, oseltamivir, zanamivir, amantadine, or rimantadine during this occurrence of ILI; or had been administered an investigational drug or investigational vaccine within 30 days prior to screening. Detailed exclusion criteria are provided in the Additional file 1.

### Sample size calculation

A sample size of 258 subjects (129 subjects in each treatment group) in the modified intent-to-treat (mITT) analysis set with a total of 430 randomly assigned subjects was calculated using the log-rank test (inputting the median survival times). Sample size calculations were performed using PASS software Version 12 (NCSS, LLC, Kaysville, Utah, USA) and considered a statistical power of 80 % to detect a clinically relevant difference between 3.5 days in the treatment group and 5 days in the placebo group. A 20 % dropout rate was also considered, which meant that a final sample size of 206 subjects (103 subjects in each treatment group) would be required for the study. However, because of the challenges faced during enrolment, which included a late influenza alert and an unexpectedly mild influenza season, the decision was made to continue enrolment until 30 April 2015. A total of 463 subjects were randomly assigned, and of these, only 137 subjects met the criteria for inclusion in the mITT analysis set.

### Statistical analysis

All analyses were conducted using SAS® software Version 9.2 (SAS Institute Inc, Cary, North Carolina, USA). All statistical tests were 2-sided hypothesis tests performed with a 5 % level of significance, which resulted in 95 % (2-sided) confidence intervals (CIs). No adjustments for multiplicity were made. The hazard ratios (HRs) and 95 % CIs were estimated using a proportional hazards model (HR >1 indicated a benefit to inosine pranobex compared with placebo). The safety analysis set consisted of all subjects who received at least 1 dose of any study drug. All analyses using the safety analysis set grouped subjects according to the treatment that the subjects had actually received. The mITT analysis set consisted of all randomly assigned subjects who had a positive laboratory confirmation of acute respiratory viral infection due to influenza A or B virus, RSV, adenovirus, or parainfluenza virus 1 or 3. This set was used for the primary and secondary efficacy analyses. Detailed primary efficacy endpoints are provided in the Additional file 1. All analyses using the mITT analysis set grouped subjects according to the randomised treatment. The intent-to-treat (ITT) analysis set included all subjects who were randomly assigned to receive double-blinded study drug and was used for the subgroup analyses.

### Randomisation and blinding

On Day 1, eligible subjects were randomly assigned to receive either inosine pranobex or placebo in a 1:1 allocation ratio with no stratification. The active drug and matching placebo tablets were provided in identical cartons that were identified with a kit number, such that all study site staff and subjects remained blinded throughout the study. Only personnel with access to the interactive web response system and clinical supplies were unblinded and had access to the treatment assignments; all other parties involved in the study were fully blinded.

### Intervention

Inosine pranobex or placebo 500-mg tablets were self-administered by the subjects for 7 days (2 tablets orally 3 times daily). The first dose was taken immediately after randomisation at the study site, and the remaining doses were to be self-administered at home. Doses were taken approximately 8 h apart but consistent with the subject’s lifestyle, ie, scheduling of dosing did not disturb the subject’s usual sleep patterns. The subjects were provided with kits containing (randomised) medication sufficient for 1 subject for 7 days of treatment. Subjects were instructed to consume no more than 42 tablets for the specified duration and were required to return the excess study drug tablets at the end-of-treatment (EOT) visit. Adherence to study drug administration was good and was monitored as part of the study.

### Procedures

The total study duration was 21 days (±3 days) for each subject and consisted of a 7-day dosing period (Day 1 to Day 7), 1 day for the EOT visit (Day 8 + 1 day), and a 13-day follow-up period (Day 21 ± 3 days) after the EOT visit. Prior to randomisation on Day 1, a nasopharyngeal swab sample was collected to test for the presence of influenza A or B virus, RSV, adenovirus, and parainfluenza virus 1 or 3 using the appropriate polymerase chain reaction analyses. The results were used to identify the subjects to be included in the mITT set and consequentially identify the subjects to be included in the primary endpoint analysis. A detailed procedure for the study visits is included in the Additional file 1.

### Efficacy and subgroup analysis

The primary efficacy endpoint was the time to resolution of all influenza-like symptoms present at baseline to none (ie, a score of 0, defined as the complete absence of symptoms, on the influenza-like symptoms assessment scale [details provided in Additional file 1]). The secondary endpoints included time to resolution of respiratory symptoms (cough, sore throat, and nasal obstruction); time to absence of fever (oral temperature of ≤37.5 °C for at least 2 consecutive readings that were at least 12 h apart); time to resumption of normal activity (ie, score of 0 on daily activities assessment scale); and frequency of viral respiratory infection complications, defined as hospitalisation, death due to ILI or complications of ILI, or requirement of antibiotic treatment for secondary bacterial infection.

A subgroup analysis was conducted for time to resolution of all influenza-like symptoms present at baseline to none in subjects with clinically diagnosed ILI. This was conducted in subjects less than 50 years of age and subjects at least 50 years of age without related ongoing disease (related ongoing disease was any medical condition with the preferred terms of asthma, bronchitis, chronic bronchitis, or chemical bronchitis that was ongoing at the start of the study). In addition, an analysis was conducted in subjects less than 50 years of age who were non-obese (BMI <30 kg/m^2^) or obese (BMI ≥30 kg/m^2^). An additional analysis was conducted for time to resolution of all influenza-like symptoms to mild or none for subjects less than 50 years of age.

### Safety analysis

Safety was evaluated during the study through analysis of adverse events (AEs), vital signs, and physical examinations.

### Bioethical issues

The study was performed in accordance with ethical principles that have their origin in the Declaration of Helsinki, International Council for Harmonization E6 (R1), and all applicable regulations. Study was approved before study start by two Multicentre Ethics Committees (MEC). One MEC in the University hospital Brno approved study for all study centres in the Czech Republic and the second one MEC of Košice Regional Office approved study for all study centres in Slovakia. All potential subjects signed an informed consent form prior to randomisation on Day 1 before any study-related procedures were performed.

## Results

**Fig. 1:**
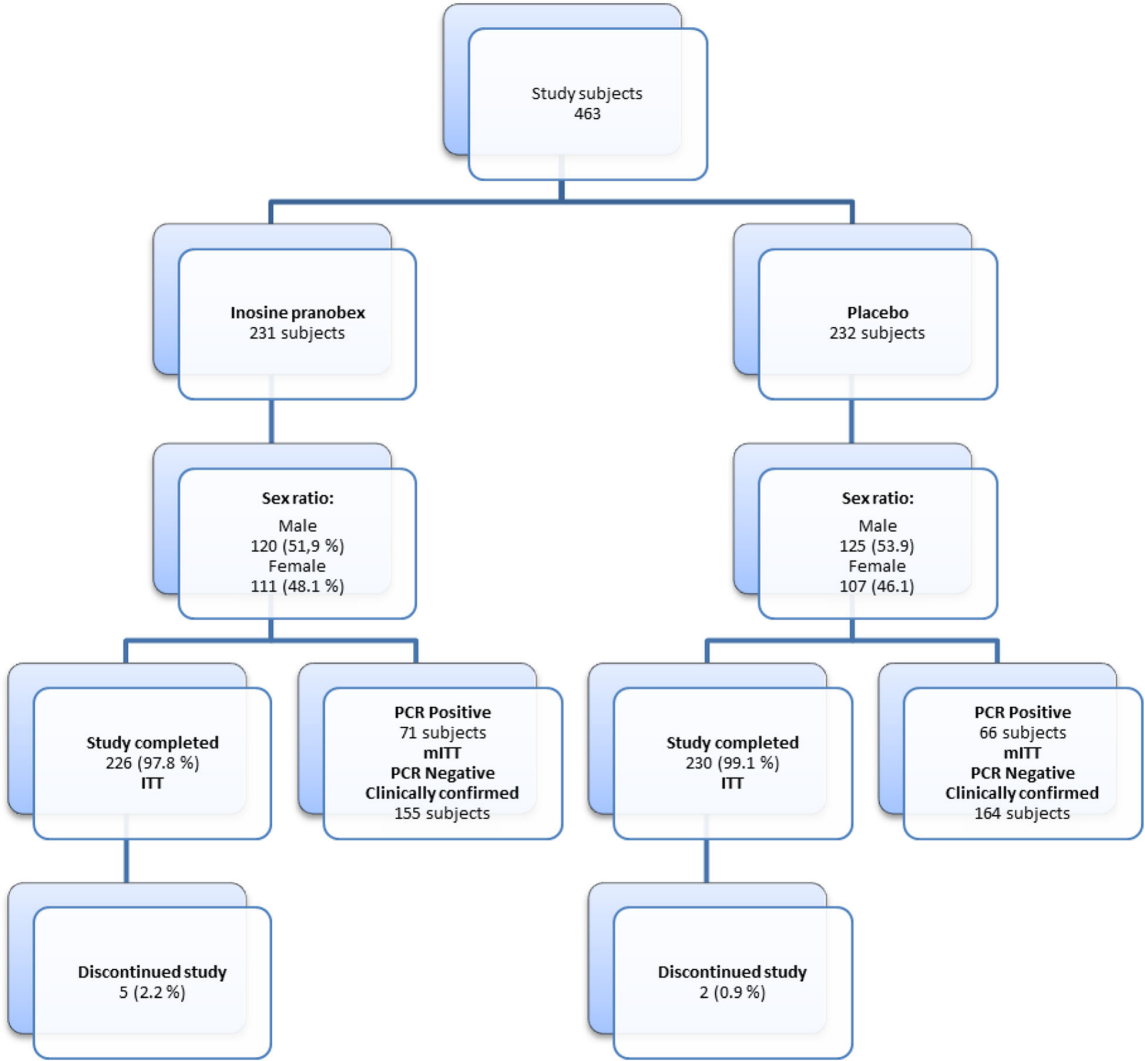
Flow-chart of enrolment, placement in treatment and placebo arms, division into the different subgroups and mITT and ITT analysis sets

The study included a total of 463 subjects who were randomly assigned to receive either inosine pranobex (*n* = 231) or placebo (*n* = 232), and 98.5 % of subjects completed the study (Fig. 1 – Flow chart). There were 7 subjects who discontinued the study; the reasons included protocol noncompliance (*n* = 2), AEs (*n* = 2; rhinopharyngitis and pleuropneumonia), and withdrawal of consent (*n* = 2). Overall, 137 subjects (29.6 %) had positive nasopharyngeal swab test results and were included in the mITT analysis set (inosine pranobex, *n* = 71; placebo, *n* = 66).

### Demographic and baseline characteristics

The details of the demographic and baseline characteristics are presented in the Additional file 1. The demographic characteristics were similar between the 2 treatment groups. The overall mean age was 41.9 years, and the overall mean BMI was 26.450 kg/m^2^ (range: 12.07 to 45.11 kg/m^2^). The majority of subjects in this study were less than 65 years of age. At baseline, most of the subjects presented with at least 1 influenza-like symptom (cough, sore throat, nasal obstruction, fatigue, headache, myalgia, or feverishness) with a score of 1, 2, or 3 in severity. Medical histories were reported for 172 subjects (74.5 %) in the inosine pranobex treatment group and 178 subjects (76.7 %) in the placebo group, and the most commonly reported medical histories were vascular disorders and surgical and medical procedures. Medical histories of gastrointestinal disorders (10.8 % in the inosine pranobex group and 11.2 % in the placebo group) and hepatobiliary disorders (10.4 % in the inosine pranobex group and 11.6 % in placebo group) were also reported.

### Compliance

Overall, the mean reported dose compliance was high, and it was similar between treatment groups (97 % in each treatment group). In the majority of subjects in both treatment groups, dose compliance ranged from 80 to 120 % (inosine pranobex, *n* = 217 [94.8 %]; placebo, *n* = 220 [93 %]).

### Efficacy analysis

**Fig. 2:**
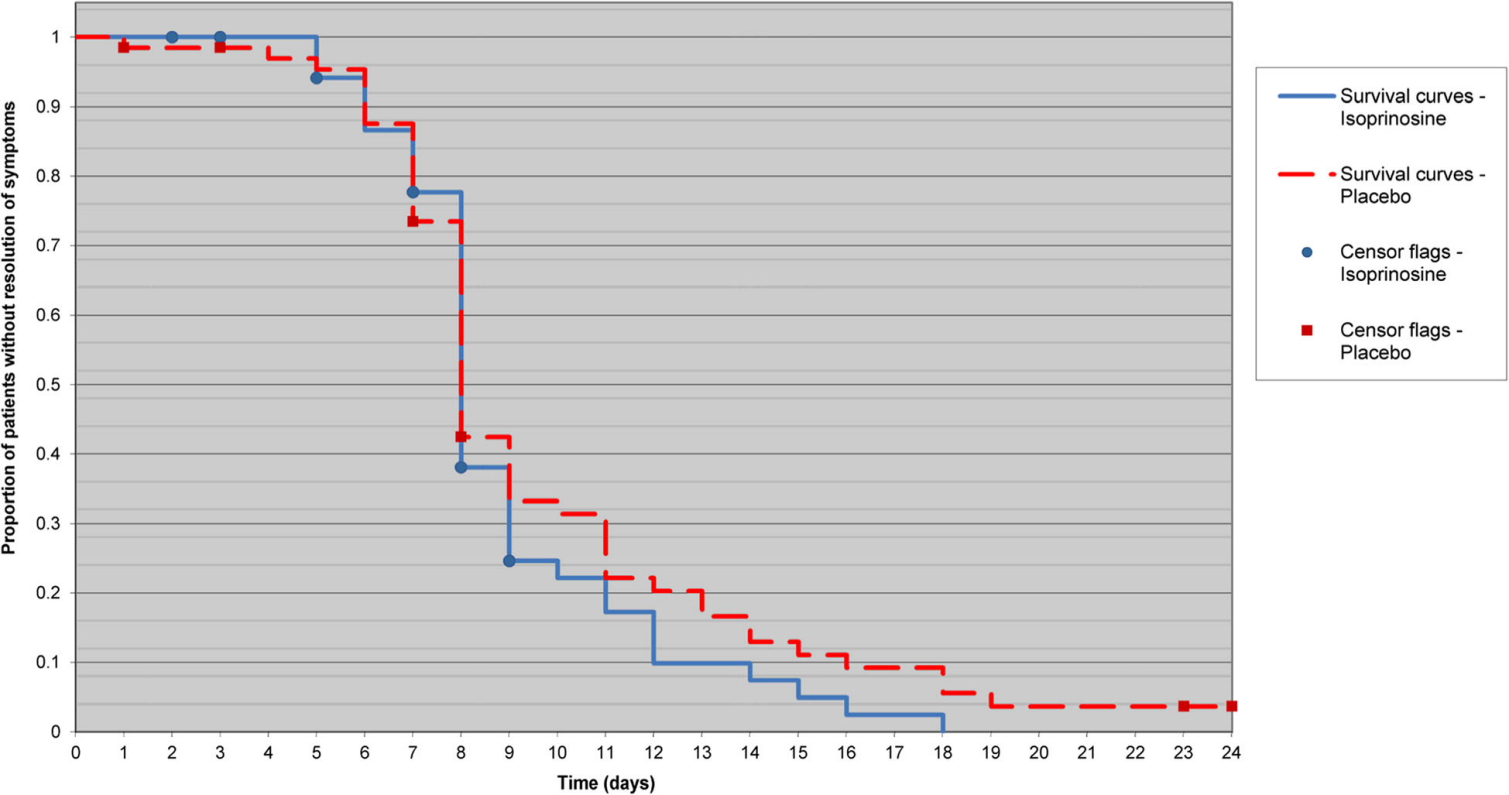
Kaplan-Meier Plot for Time to Resolution of All Influenza-Like Symptoms. (mITT Analysis Set). mITT = modified intent-to treat analysis set. Note: Time to resolution was the total number of days from randomisation to the first instance at which all influenza-like symptoms had a score of 0 (date of resolution of all influenza-like symptoms minus the date of randomisation + 1)

The difference in time to resolution of all influenza-like symptoms between treatment groups was not statistically significant but showed a trend towards improvement in subjects in the inosine pranobex group compared with subjects in the placebo group (HR: 1.175; 95 % CI: 0.806, 1.714; *p* = 0.324) (Fig. 2). A substantial decrease in the proportion of subjects with all influenza-like symptoms was observed in the inosine pranobex group after 9 days while a decrease to similar proportions occurred only after 11 days for subjects in the placebo group.

The differences in time to resolution for the secondary endpoints also showed a similar trend towards improvement for subjects in the inosine pranobex group compared with subjects in the placebo group, but these secondary endpoint differences were not significant. The detailed results are provided in the Additional file 1.

### Subgroup analysis

**Fig. 3:**
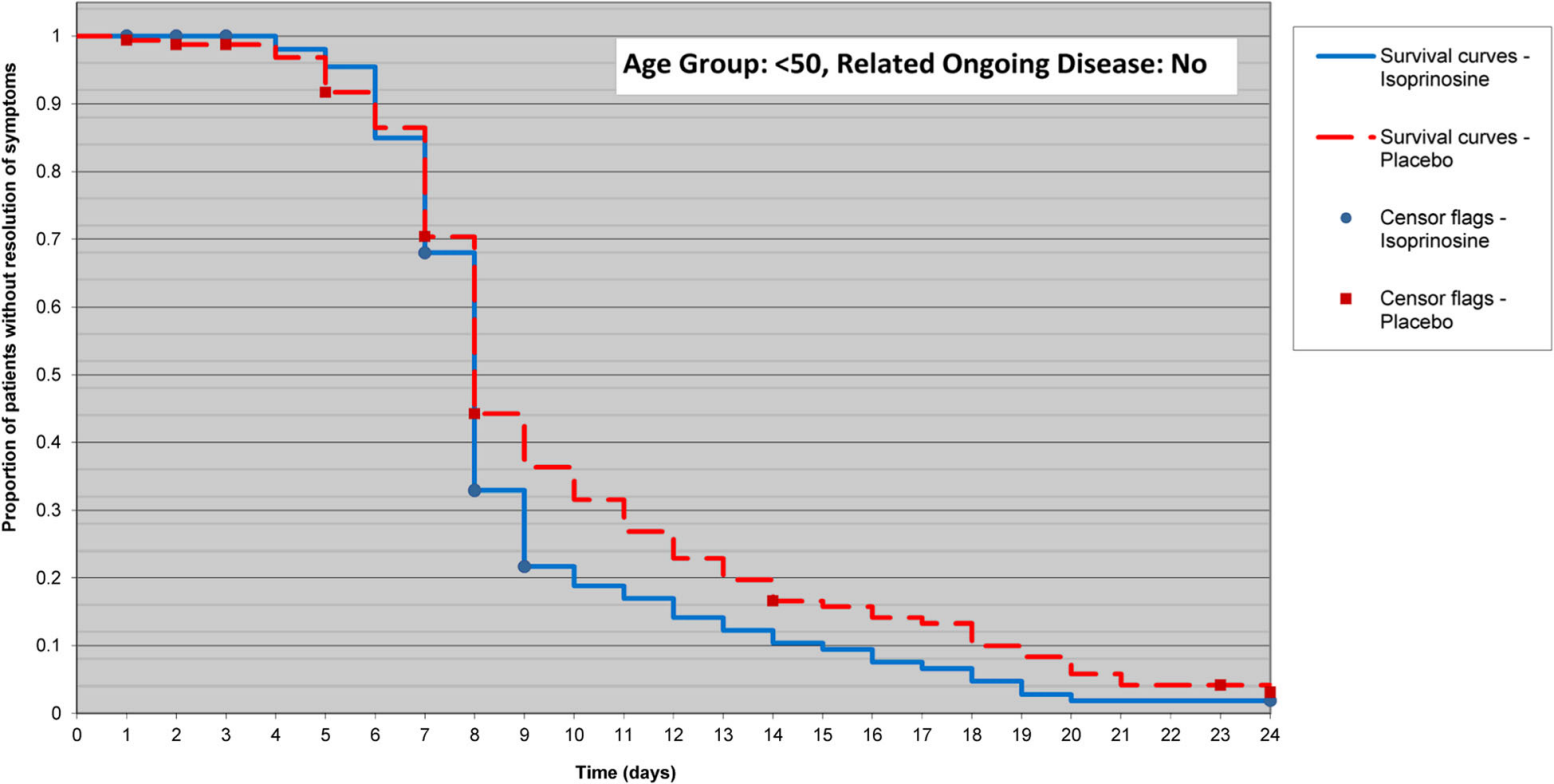
Time to Resolution of Influenza-Like Symptoms in Subjects <50 Years Without Related Ongoing Disease. Analysis carried out in the intent-to treat analysis set. Note: Time to resolution was the total number of days from randomisation to the first instance at which all influenza-like symptoms had a score of 0 (date of resolution of all influenza-like symptoms minus the date of randomisation + 1)

In the subgroup analysis, for subjects less than 50 years of age without related ongoing disease, the difference in time to resolution of all influenza-like symptoms between treatment groups was statistically significant (*p* = 0.050) and showed faster improvement in subjects in the inosine pranobex group compared with subjects in the placebo group (HR: 1.234; 95 % CI: 0.969, 1.571) (Fig. 3 and Table 1). However, for subjects at least 50 years of age without related ongoing disease, the difference in time to resolution of all influenza-like symptoms between treatment groups was not statistically significant (HR:0.887; 95 % CI: 0.0.604, 1.303; *p* = 0.488).

**Table 1:** Time to resolution of all influenza-like symptoms between treatment groups

*Group for analysis*	*Hazard ratio (HR)*	*95 % CI*	*P-value*
*Age Group: <50 and BMI <30*	***1.307***	***1.010–1.691***	***0.018***
*Age Group: <50 and BMI ≥30*	*0.782*	*0.429–1.426*	*0.370*
*Age Group: ≥50 and BMI <30*	*0.838*	*0.534–1.316*	*0.383*
*Age Group: ≥50 and BMI ≥30*	*0.879*	*0.445–1.736*	*0.683*
*Age Group: <50 and resolution of symptoms to "mild" or "none"*	***1.298***	***1.035–1.627***	***0.009***

The "bold data" are statisticly significant

For non-obese (BMI <30 kg/m^2^) subjects less than 50 years of age, the difference in time to resolution of all influenza-like symptoms between treatment groups was statistically significant (*p* = 0.018) and showed a faster improvement in subjects in the inosine pranobex group compared with subjects in the placebo group (HR: 1.307; 95 % CI: 1.010, 1.691). However, for obese (BMI ≥30 kg/m^2^) subjects less than 50 years of age, the difference in time to resolution of all influenza-like symptoms between treatment groups was not statistically significant (HR: 0.782; 95 % CI: 0.429, 1.426; *p* = 0.370).

In subjects less than 50 years of age, the difference in time to resolution of all influenza-like symptoms to mild or none between treatment groups was statistically significant (*p* = 0.009) and showed a faster improvement in subjects in the inosine pranobex group compared with subjects in the placebo group (HR: 1.298; 95 % CI: 1.035, 1.627).

### Safety analysis

The proportion of subjects experiencing treatment-emergent AEs (TEAEs) and the number of TEAEs were lower in the inosine pranobex group (39 subjects [17.0 %], 55 TEAEs) than in the placebo group (48 subjects [20.4 %], 72 TEAEs). The most common TEAEs (reported in more than 3 subjects in either treatment group) were bacterial superinfection (inosine pranobex, *n* = 10 [4.4 %]; placebo, *n* = 2 [0.9 %]) and diarrhoea (inosine pranobex, *n* = 2 [0.9 %]; placebo, *n* = 7 [3.0 %]). No other TEAE was reported in more than 3 subjects in either treatment group.

The majority of TEAEs were mild or moderate in severity and were unrelated to study drug, in the opinion of the investigator. Overall, 4 severe TEAEs were reported in 3 subjects (0.6 %). Severe TEAEs of rhinopharyngitis, maxillary sinusitis, and vertebrogenic pain syndrome were reported in 1 subject (0.4 %) each in the inosine pranobex group, and the severe TEAE of pleuropneumonia was reported in 1 subject (0.4 %) in the placebo group. No deaths were reported during the study.

Overall, 6 subjects (2.6 %) in the inosine pranobex group and 7 subjects (3.0 %) in the placebo group experienced TEAEs that led to study drug discontinuation. Three treatment-emergent serious AEs (SAEs) were reported in 2 subjects (0.4 %). Severe rhinopharyngitis and severe vertebrogenic syndrome were reported in 1 subject in the inosine pranobex group, and 1 subject in the placebo group reported severe pleuropneumonia. These SAEs resulted in permanent discontinuation of the study drug and discontinuation of both subjects from the study. All SAEs resolved, and none of the SAEs in the opinion of the investigator were related to the study drug.

The mean changes in vital sign and physical examination values from baseline were small, and no clinically significant trends were observed between treatment groups. No pregnancies were reported during the study.

## Discussion

This was a Phase 4, randomised, placebo-controlled, double-blind, multicentre study that evaluated the efficacy of inosine pranobex in subjects with clinically diagnosed ILI, including subjects with laboratory-confirmed acute respiratory viral infections due to influenza A or B virus, RSV, adenovirus, or parainfluenza virus 1 or 3. The study also evaluated the efficacy of inosine pranobex in subgroups of subjects less than 50 years of age who were without related diseases (such as asthma, bronchitis, chronic bronchitis, and chemical bronchitis) and who were non-obese (BMI <30 kg/m^2^) or obese (BMI ≥30 kg/m^2^) as well as a subgroup of subjects at least 50 years of age without related ongoing disease. In addition, a subgroup analysis was conducted in subjects less than 50 years of age for time to resolution of all influenza-like symptoms to mild or none.

In the current study, the analysis of the primary endpoint of time to resolution of all influenza-like symptoms showed a faster improvement in subjects treated with inosine pranobex compared with subjects administered placebo, although the difference between treatment groups did not reach the threshold of statistical significance. The results were similar for the secondary efficacy endpoints of time to resolution of respiratory symptoms (cough, sore throat, and nasal obstruction), time to absence of fever, and time to resumption of normal activity. The difference in the occurrence of viral respiratory infection complications between treatment groups was not statistically significant.

Immunosenescence, ie, the age-related decline of the immune system, and obesity play an important role in the efficacy of the immune response to pathogens [18, 19]. Older subjects show a diminished immune response to pathogens, which increases their risk for severe infection and compromises their ability to adequately combat viral infections. This phenomenon was observed with split-virus influenza vaccines; a low response to the vaccine was observed in older adults, whereas the vaccine was effective in younger subjects. This low response resulted in increased susceptibility to influenza and associated complications in older adults compared to younger adults who typically benefit from a higher response [20, 21]. Obesity has also been identified as an independent risk factor for increased susceptibility to influenza virus infection; this susceptibility results from diminished CD4+ and CD8+ T-cell responses and lower influenza vaccine antibody levels [19, 22, 23]. Obesity may also increase the risk of pneumonia or other infections by restricting lung volume [24]. Immunosenescence and obesity can bias efficacy studies because of the impaired response of the immune system to pathogens, as the risk of complications is increased in such individuals.

In the subgroup analysis of the current study, in subjects less than 50 years of age who were without related ongoing disease and in those less than 50 years of age who were non-obese (BMI <30 kg/m^2^), statistically significant differences in time to resolution of influenza-like symptoms favoured the inosine pranobex group over the placebo group. Statistically significant differences were not observed between the inosine pranobex and placebo groups in subjects at least 50 years of age without related ongoing disease or in subjects less than 50 years of age who were obese (BMI ≥30 kg/m^2^). Thus, the efficacy of inosine pranobex was improved in non-obese subjects compared with obese subjects, probably because the immune system in the former is more capable of defending against pathogens and is not negatively affected by obesity-related complications. Older patients have a decreased immune response to pathogens as a result of immunosenescence; therefore, they may take longer to recover from illnesses such as influenza and anti-influenza drugs may not be as effective. In an additional analysis, in subjects less than 50 years of age, statistically significant differences in time to resolution of influenza-like symptoms to mild or none favoured the inosine pranobex group over the placebo group, thus indicating that the improvement of symptoms was better with inosine pranobex than placebo in this subset of subjects (HR: 1.298; 95 % CI: 1.035, 1.627).

A substantial decrease in the proportion of subjects with all influenza-like symptoms was observed in the inosine pranobex group after 9 days while a decrease to similar proportions occurred only after 11 days for subjects in the placebo group. This difference could be a result of the time necessary for activation of the immune system, as inosine pranobex acts indirectly by stimulating the immune system and does not directly attenuate the symptoms. This result is also consistent with the results observed in a study in healthy volunteers in which inosine pranobex showed immunomodulating properties through an increase in serum levels of interferon-γ, IL-2, IL-10, and tumour necrosis factor-α from 7 to 10 days [13].

The approved dose and treatment duration of inosine pranobex (two 500-mg tablets orally 3 times daily) were used in this study, and the administered treatment did not vary according to weight or symptom duration. From the subgroup analysis, the posology of inosine pranobex (3 g/day orally) in this study is most suitable for subjects less than 50 years of age without related ongoing disease and subjects less than 50 years of age who were non-obese (BMI <30 kg/m^2^). For certain subjects, such as those at least 50 years of age and those who are obese, different dosing strategies could be more appropriate; varying the dosing regimen requires further evaluation.

The use of a placebo-only group in this study was justified because ILI is generally mild and self-limiting and no other treatments are approved for acute respiratory viral infections other than influenza. In addition, the use of influenza-specific antivirals (neuraminidase inhibitors or amantadine) is not a component of routine medical management of ILI in many countries, including the countries in which the study was conducted.

Performing a high-quality efficacy trial for ILI is challenging because of epidemiologic considerations from the influenza outbreak period; the influenza season cannot be predicted in advance and can vary from year to year [25]. Furthermore, the enrolment of subjects with symptoms that have been present for less than 36 h is necessary, as the first 36 h is the period of maximal viral replication and antiviral medication is expected to have the most benefit during this time. The current study was anticipated to be performed in 1 influenza season in the Northern Hemisphere, between 01 October 2014 and 30 April 2015, and enrol the required number of subjects in each group during this timeframe. However, enrolment was challenging, as it could not be commenced because of a low attack rate and was only started after a late alert was issued by national public health authorities in the first week of December 2014 regarding the statistically higher incidence of acute respiratory viral infections. A low density of circulating viruses was present until the end of January 2015, which resulted in the enrolment of subjects until 30 April 2015 in order to maximize the number of completed subjects. Approximately 60 % of the randomised subjects were expected to have a positive laboratory confirmation of acute respiratory tract infection. However, only 137 subjects met the criteria for inclusion in the mITT analysis set, which was 121 subjects fewer than the 258 subjects expected. The effects of the absence of a significant influenza outbreak adversely affected the statistical power, thus reducing the power of the study, which could potentially explain the lack of statistical significance for the primary and secondary efficacy endpoints.

A slightly longer duration of treatment or a different dosing strategy may have influenced the observed efficacy of inosine pranobex, as proof of therapeutic effect requires a high attack rate, ie, a higher number of sick patients during one influenza season. The attack rate is difficult to predict, and studies, which will adjust the sample size calculations to account for the possibility of a below-average flu season or studies with longer duration, e.g., those that include 2 or more influenza seasons to account for lower than predicted attack rates, are necessary to achieve the desired results [25].

In addition, 10.8 % of enrolled subjects in the inosine pranobex group reported a medical history of gastrointestinal disorders and 10.4 % reported hepatobiliary disorders. Pharmacokinetic parameters were not measured in this study, but it is possible that the presence of these disorders at baseline may have affected the absorption, distribution, and metabolism of the study drug and may have influenced the study results, including the efficacy results. Furthermore, age, comorbidities, and the obesity of enrolled subjects, particularly in subjects more than 50 years of age, may also have affected the outcome of the study.

The safety analysis demonstrated that inosine pranobex treatment was well tolerated, and no major differences in safety profiles were observed between treatment groups. Treatment-emergent SAEs were reported in 2 subjects, and none were considered to be related to study drug by the investigators. No subjects died during the study. No significant changes were observed in vital signs and physical examinations in either study group.

## Conclusions

The study results indicate the safety of inosine pranobex for the treatment of subjects with confirmed acute respiratory viral infections and confirms the efficacy of inosine pranobex versus placebo in healthy non-obese subjects less than 50 years of age with clinically diagnosed influenza-like illnesses. The results of this study were affected by epidemiologic considerations, which included a late influenza alert and a low density of circulating viruses. Further studies may be important to define predictors of treatment success, including the potential of different dosing strategies in certain patient populations, such as those with underlying conditions that may impact drug plasma levels and related drug effects.

## Additional file

Additional file 1: The primary efficacy assessment; Secondary Efficacy Endpoints; Detailed primary endpoint; Inclusion and exclusion criteria; Procedures and **Table S1.** Influenza-Like Symptoms Assessment Scale; **Table S2.** Demographic and Other Baseline Characteristics (ITT Analysis Set); Secondary endpoints; **Table S3.** Time to Resolution of Respiratory Symptoms and Resumption of Normal Activity (mITT Analysis Set); **Table S4.** Treatment-Emergent adverse events (Safety analysis set); **Table S5.** Treatment-Emergent AE occurring in at least 3 % of subjects overall by system organ class per treatment (Safety analysis set); **Figure S1.** Flow-chart of enrolment, placement in treatment and placebo arms, and division into the different subgroups for analysis; **Table S6.** Time to resolution of all influenza-like symptoms between treatment groups; **Table S7.** Time to Resolution of All Influenza-like Symptoms (ITT Analysis Set), Age Group: <50 and BMI <30; **Table S8.** Time to Resolution of All Influenza-like Symptoms (ITT Analysis Set), Age Group: <50; Statement: Patient data are provided as summary data in anonymized form. (DOC 504 kb)

## Abbreviations

AE: Adverse event
BMI: Body mass index
CI: Confidence interval
EOT: End of treatment
HR: Hazard ratio
IL: Interleukin
ILI: Influenza-like illnesses
ITT: Intent-to treat
MEC: Multicentre ethics committees
mITT: Modified intent to treat
RSV: Respiratory syncytial virus
SAE: Serious AE
TEAE: Treatment-emergent adverse event
