# Supplementary material for: Inosine pranobex is safe and effective for the treatment of subjects with confirmed acute respiratory viral infections: analysis and subgroup analysis from a Phase 4, randomised, placebo-controlled, double-blind study
Source: BMC Infect Dis. 2016 Nov 7;16:648. doi: 10.1186/s12879-016-1965-5 (PMC5100179; doi:10.1186/s12879-016-1965-5)
Supplement: Additional file 1: — The primary efficacy assessment; Secondary Efficacy Endpoints; Detailed primary endpoint; Inclusion and exclusion criteria; Procedures and Table S1. Influenza-Like Symptoms Assessment Scale; Table S2. Demographic and Other Baseline Characteristics (ITT Analysis Set); Secondary endpoints; Table S3. Time to Resolution of Respiratory Symptoms and Resumption of Normal Activity (mITT Analysis Set); Table S4. Treatment-Emergent adverse events (Safety analysis set); Table S5. Treatment-Emergent AE occurring in at least 3 % of subjects overall by system organ class per treatment (Safety analysis set); Figure S1. Flow-chart of enrolment, placement in treatment and placebo arms, and division into the different subgroups for analysis; Table S6. Time to resolution of all influenza-like symptoms between treatment groups; Table S7. Time to Resolution of All Influenza-like Symptoms (ITT Analysis Set), Age Group: <50 and BMI <30; Table S8. Time to Resolution of All Influenza-like Symptoms (ITT Analysis Set), Age Group: <50; Statement: Patient data are provided as summary data in anonymized form. (DOC 504 kb) [file 12879_2016_1965_MOESM1_ESM.doc]

**Additional File for**

**Inosine pranobex is safe and effective for the treatment of subjects with confirmed acute respiratory viral infections: analysis and subgroup analysis from a Phase 4, randomized, placebo-controlled double-blind study**

Jiri Beran^1^, Eva Salapova^2^, Marian Spajdel^3, 4^ on behalf of the Isoprinosine Study (EWO‑ISO-2014/1) Team Primary Efficacy Endpoint

**The primary efficacy assessment** for comparison between inosine pranobex and placebo was the time to resolution of all influenza-like symptoms present at baseline to none (i.e. score of 0 on influenza-like symptoms assessment scale).

**Secondary Efficacy Endpoints**

The secondary efficacy endpoints of this study included the following:

- Time to resolution of all constitutional symptoms present at baseline to none (i.e. score of 0 on influenza-like symptoms assessment scale)
- Time to resolution of cough present at baseline to none (i.e. score of 0 on influenza like symptoms assessment scale)
- Time to resolution of sore throat present at baseline to none (i.e. score of 0 on influenza like symptoms assessment scale)
- Time to resolution of nasal obstruction present at baseline to none (i.e. score of 0 on influenza-like symptoms assessment scale)
- Time to absence of fever (oral temperature of ≤37.5°C for at least 2 consecutive readings at least 12 hours apart)
- Time to resumption of normal activity (i.e. score of 0 on daily activities assessment scale)
- Frequency of viral respiratory infection complications of hospitalisation, death due to influenza like illness or complications of influenza like illness, and requirement for antibiotics to treat secondary bacterial infections.

# Inclusion Criteria

A subject was eligible for inclusion if he/she met all of the following criteria:

1. Was male or nonpregnant female subject aged 18 to 75 years; female subject was either surgically sterile, postmenopausal (no menses during the previous 12 months), or was practicing an effective method of birth control as determined by the investigator (eg, oral contraceptives, double barrier methods, injectable or implanted hormonal contraceptives, tubal ligation, or male partner with vasectomy or complete abstinence).
2. Had an influenza-like illness defined as:

- oral temperature of ≥38°C obtained at study site. If oral temperature measured at screening was <38°C, then the subject was still eligible if the self-measured temperature at home within 12 hours prior to screening was either an oral temperature of ≥38°C or an axillary temperature of >37.5°C and the subject had taken an antipyretic within 4 hours prior to screening,
- at least 1 of the following respiratory symptoms: cough, sore throat, or nasal obstruction that were considered by the subject to be moderate or severe in intensity (a score of more than 1 on a 4-point scale), and
- at least 1 of the following constitutional symptoms: fatigue, headache, myalgia, or feverishness that were considered by the subject to be moderate or severe in intensity (a score of more than 1 on a 4-point scale).

1. Had onset of influenza-like illness no more than 36 hours prior to screening, defined as when the subject experienced fever and at least one respiratory symptom and at least one constitutional symptom.
2. Was capable of understanding the written informed consent, provided signed and witnessed written informed consent, and agreed to comply with protocol requirements, including completion of the subject diary.

# Exclusion Criteria

Subject was to be excluded from participation in this study if he/she met any of the following criteria:

1. Had immunosuppressive disorder or who was receiving immunosuppressive therapy (immunosuppressants, antitumour agents, etc).
2. Was receiving treatment with xanthine oxidase inhibitors (allopurinol) or uricosuric agents, or treatment with thiazide diuretics (eg, hydrochlorothiazide, chlorthalidone, and indapamide) or loop diuretics (eg, furosemide, torsemide, and ethacrynic acid).
3. Had chronic renal dysfunction (creatinine clearance <50 mL/min).
4. Had liver disorder (severe liver function impairment; aspartate aminotransferase and alanine aminotransferase values greater than 3 times the upper limit of normal).
5. Was lactose intolerant.
6. Had cancer in a nonremission stage (subject with nonmetastatic basal cell or squamous cell skin cancer or other early cancer for which surgical resection was considered to be completely curative was eligible for enrolment).
7. Was a resident of a nursing home or other long-term care institution.
8. Was receiving treatment with zidovudine.
9. Was pregnant or lactating/breastfeeding female.
10. Had received any dose of inosine pranobex, oseltamivir, zanamivir, amantadine, or rimantadine during this period of influenza-like illness.
11. Had received prior treatment with any investigational drug or vaccine within 30 days prior to screening.
12. Had known hypersensitivity to inosine acedoben dimepranol or any of the excipients comprising inosine pranobex from the 500 mg inosine pranobex tablets.
13. Had a medical history of hyperuricaemia or gout.
14. Was unable to take oral medications.
15. Had any preexisting illness that, in the opinion of the investigator, would place the subject at an increased risk through participation in this study.
16. Was a subject who, in the judgment of the investigator, to be unlikely to comply with the requirements of this protocol.

# Procedures

On Day 1, prior to randomisation, the subjects who had consented to participate in the study completed the screening procedures, which included the collection of demographic details and other baseline characteristics and an assessment of influenza‑like symptoms on a 4‑point scale (Table 1).

Table 1: Influenza-Like Symptoms Assessment Scale

| Score | Severity | Definition |
| --- | --- | --- |
| 0 | None | Complete absence of symptoms |
| 1 | Mild | Slight |
| 2 | Moderate | Definitely present |
| 3 | Severe | Marked, intense |

The subjects were randomly assigned to either inosine pranobex or placebo and were to continue dosing for 7 days. On Day 1, subjects were provided with diary cards to record the following: study drug administration (dosing times), each time they took the study drug; nonroutine concomitant medications, at each time of administration; influenza‑like illness respiratory and constitutional symptoms, once daily in the evening (between 18:00 and 22:00 hours); ability to perform daily activities, once daily in the evening (between 18:00 and 22:00 hours) using the 4‑point daily activities assessment scale (0 = no effect on activities; 1 = able to do all normal activities with effort; 2 =some impact on activities, able to attend work or education; 3 =unable to carry out normal activities, unable to attend work or education); and oral temperature, 3 times a day. Diary cards were completed for symptoms from the evening of Day 1 until the end-of-treatment visit even if all of the symptoms resolved.

At the end-of-treatment visit (Day 8 + 1 day), each subject returned to the study site with his or her diary cards and unused study drug, and each subject was evaluated for complications associated with viral respiratory infection. A follow-up telephone call was conducted on Day 21 (± 3 days) to assess viral respiratory infection-associated complications, for all subjects

# Demographic and Baseline Characteristics

Table 2: Demographic and Other Baseline Characteristics (ITT Analysis Set)

|  | **Treatment** | | **Total**  **(N = 463)** |
| --- | --- | --- | --- |
|  | **Inosine Pranobex**  **(N = 231)** | **Placebo**  **(N = 232)** |  |
| Mean (SD) age, years | 41.9 (12.86) | 41.8 (13.45) | 41.9 (13.15) |
| Age group, n (%) |  |  |  |
| 18-40 years | 116 (50.2) | 118 (50.9) | 234 (50.5) |
| 41-64 years | 103 (44.6) | 101 (43.5) | 204 (44.1) |
| ≥65 years | 12 (5.2) | 13 (5.6) | 25 (5.4) |
| Sex, n (%) |  |  |  |
| Male | 120 (51.9) | 125 (53.9) | 245 (52.9) |
| Female | 111 (48.1) | 107 (46.1) | 218 (47.1) |
| Fertility status (female only), n^a^ (%) |  |  |  |
| Premenarche | 0 | 0 | 0 |
| Sterile | 5 (2.2) | 4 (1.7) | 9 (1.9) |
| Postmenopausal | 30 (13.0) | 27 (11.6) | 57 (12.3) |
| Potentially able to bear children | 76 (32.9) | 76 (32.8) | 152 (32.8) |
| Mean (SD) weight, kg | 79.04 (16.530) | 80.00 (16.104) | 79.52 (16.307) |
| Mean (SD) height, cm | 173.1 (9.21) | 173.4 (9.29) | 173.2 (9.24) |
| Mean (SD) BMI, kg/m^2^ | 26.360 (5.0207) | 26.540 (4.6547) | 26.450 (4.8364) |
| Subjects with at least 1 concomitant medication, n (%) | 222 (96.9) | 227 (96.6) | 449 (96.8) |
| Subjects with at least 1 prior medication, n (%) | 127 (55.5) | 151 (64.3) | 278 (59.9) |
| Subjects with any medical history, n (%) | 172 (74.5) | 178 (76.7) | 350 (75.6) |
| Abbreviations: BMI, body mass index; ITT, intent to treat; SD, standard deviation  ^a^ Percentage expressed as percentage of total patients; ^b^ Related ongoing disease was any medical condition with the preferred terms of asthma, bronchitis, chronic bronchitis, or chemical bronchitis that was ongoing at the start of the study | | | |

# Secondary Endpoints

In the modified intent-to-treat analysis set, the times to resolution of respiratory symptoms (cough, sore throat and nasal obstruction), times to absence of fever, and times to resumption of normal activity were similar between treatment groups. The differences in time to resolution were not statistically significant but showed a trend towards improvement in subjects in the inosine pranobex group compared with subjects in the placebo group, but these secondary endpoint differences were not significant (Table 3).

**Table 3**: Time to Resolution of Respiratory Symptoms and Resumption of Normal Activity (mITT Analysis Set)

| **Endpoints** | **HR* (95%CI)**  **(Inosine Pranobex/Placebo)** | **p Value** |
| --- | --- | --- |
| Time to resolution of cough | 1.201 (0.822, 1.754) | 0.275 |
| Time to resolution of sore throat | 1.051 (0.704, 1.568) | 0.783 |
| Time to resolution of nasal obstruction | 1.060 (0.732, 1.535) | 0.723 |
| Time to absence of fever | 1.148 (0.814, 1.618) | 0.268 |
| Time to resumption of normal activity | 1.038 (0.718, 1.502) | 0.819 |

Abbreviations: CI, confidence interval; HR, hazard ratio; mITT, modified intent to treat.
*HR>1 indicates a benefit to inosine pranobex.

The numbers of subjects who experienced at least one viral respiratory infection complication, which included hospitalisation, death due to influenza-like illness or complications of influenza-like illness, or the requirement of antibiotic treatment for secondary bacterial infection, were similar between the treatment groups (Inosine pranobex = 6 subjects [8.5%]; placebo = 5 subjects [7.6%]), and the difference between treatment groups was not statistically significant (p = 0.831).

4

5

Figure-1: Flow-chart of enrolment, placement in treatment and placebo arms, and division into the different subgroups for analysis


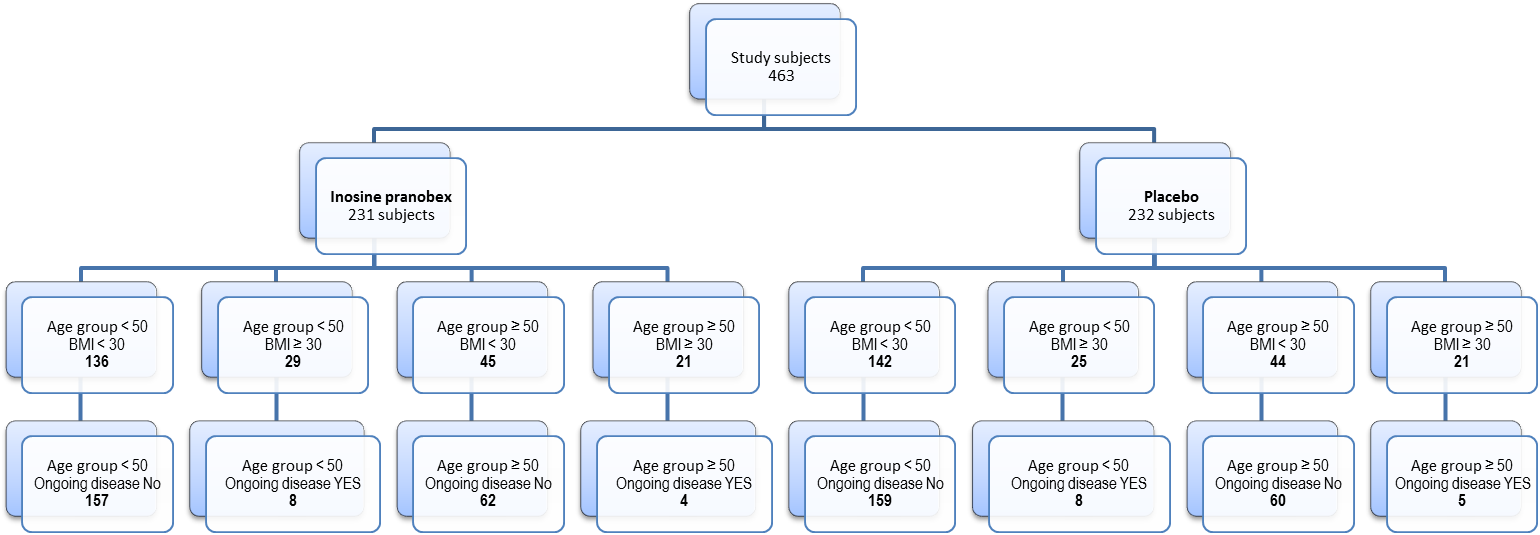


**Table-6**: Time to resolution of all influenza-like symptoms between treatment groups

| **Group for analysis** | **Hazard ratio (HR)** | **95 % CI** | **P-value** |
| --- | --- | --- | --- |
| Age Group: <50 and BMI <30 | **1.307** | **1.010 - 1.691** | **0.018** |
| Age Group: <50 and BMI ≥30 | 0.782 | 0.429 - 1.426 | 0.370 |
| Age Group: ≥50 and BMI <30 | 0.838 | 0.534 - 1.316 | 0.383 |
| Age Group: ≥50 and BMI ≥30 | 0.879 | 0.445 - 1.736 | 0.683 |
| Age Group: <50 and resolution of  symptoms to "mild" or "none" | **1.298** | **1.035 - 1.627** | **0.009** |

Table 7 Time to Resolution of All Influenza-like Symptoms (ITT Analysis Set)

| **Age Group: <50**  **BMI <30** | Treatment | |
| --- | --- | --- |
|  | Isoprinosine  (N=136) | Placebo  (N=142) |
| Number of events – n (%) | 118 (86.7) | 122 (85.9) |
| Number of censored observations – n (%) | 18 (13.2) | 20 (14.1) |
| Time to resolution - days |  |  |
| Lower quartile | 7.0 | 7.0 |
| Median | 8.0 | 8.0 |
| Upper quartile | 10.0 | 12.0 |
| P value | 0.018 |  |
| Hazard ratio | 1.307 |  |
| 95% CI | 1.010, 1.691 |  |
| Abbreviation: CI, confidence interval; NC, not calculated.  Time to resolution was calculated as the total number of days from randomisation to first instance where all influenza-like symptoms had a score of 0 (date of resolution of all influenza‑like symptoms minus date of randomisation + 1). The hazard ratio and 95% CI were estimated using a proportional hazards model (Hazard ratio > 1 indicated a benefit to Isoprinosine). All other values were estimated using a log-rank test. | | |

**Table 8** Time to Resolution of All Influenza-like Symptoms (ITT Analysis Set)

| **Age Group: <50** | **Treatment** | |
| --- | --- | --- |
|  | **Isoprinosine**  **(N=164)** | **Placebo**  **(N=167)** |
| Number of events – n (%) | 151 (92.1) | 155 (92.8) |
| Number of censored observations – n (%) | 13 (7.9) | 12 (7.2) |
| Time to resolution - days |  |  |
| Lower quartile (95% CI) | 5.0 (5.0, 6.0) | 6.0 (6.0, 7.0) |
| Median (95% CI) | 7.0 (NC, NC) | 7.0 (7.0, 8.0) |
| Upper quartile (95% CI) | 8.0 (8.0, 10.0) | 9.0 (8.0, 12.0) |
| P value | 0.009 |  |
| Hazard ratio | 1.299 |  |
| 95% CI | 1.035, 1.627 |  |
| Abbreviation: CI, confidence interval; NC, not calculated.  Time to resolution was calculated as the total number of days from randomisation to first instance where all influenza-like symptoms had a score of 0 (date of resolution of all influenza‑like symptoms minus date of randomisation + 1). The hazard ratio and 95% CI were estimated using a proportional hazards model (Hazard ratio > 1 indicated a benefit to Isoprinosine). All other values were estimated using a log-rank test. | | |
|  | | |
